# Exosome-transmitted circCOG2 promotes colorectal cancer progression via miR-1305/TGF-β2/SMAD3 pathway

**DOI:** 10.1038/s41420-021-00680-0

**Published:** 2021-10-11

**Authors:** Lei Gao, Xiaolong Tang, Qingsi He, Guorui Sun, Chao Wang, Hui Qu

**Affiliations:** 1 Department of General Surgery, The First Affiliated Hospital of Harbin Medical University, Harbin, China; 2 Department of General Surgery, Qilu Hospital of Shandong University, Jinan, China

**Keywords:** Colorectal cancer, Non-coding RNAs

## Abstract

Circular RNAs (circRNA) are abundantly present in the exosome. Yet, the role of exosome-transmitted circRNA in colorectal cancer (CRC) remains unclear. In this study, we examined the function and mechanism of circCOG2 in CRC. We analyzed the expression of circCOG2 in CRC tissues, plasmas, and exosomes by qRT-PCR. The function of circCOG2 was evaluated by CCK-8, clone formation, transwell and wound healing assay, and using an in vivo study; while its mechanism was analyzed using a dual luciferase reporter assay, RNA pull-down assay, Western blot, and rescue experiments. We found that circCOG2 was increased in CRC tissues, plasmas, and exosomes. Upregulated circCOG2 promoted CRC proliferation, migration, and invasion through the miR-1305/TGF-β2/SMAD3 pathway, and this effect could be transmitted from CRC cells with the high metastatic potential to CRC cells with low metastatic potential by exosomes. Our results revealed that circCOG2 is correlated with poor prognosis and may be used as a therapeutic target for CRC.

## Introduction

Colorectal cancer is the fourth leading cause of death and the third most commonly diagnosed cancer worldwide [1]. Despite the fact that great advances have been made in the treatment, the prognosis of CRC patients remains poor. Tumor metastasis is the main cause of death in CRC patients, with a five-year overall survival (OS) of 20% [1]. Tumor metastasis is a multi-step process that involves multiple molecules; however, the exact mechanism underlying each process remains unclear.

Circular RNA (circRNA), an endogenous non-coding RNA generated by precursor mRNA back-splicing mechanism [2], is abundant and stable in eukaryotic cells. circRNA regulates the transcription and translation, interacts with RNA-binging proteins, and may act as miRNA sponge [3, 4]. A growing number of studies have revealed that circRNA is involved in CRC progression. Chen et al. demonstrated that hsa_circ_101555 promotes CRC proliferation [5]. While Hsa_circ_001680 [6] and circRNA CCDC66 [7] promoted CRC metastasis. This evidence illustrated the important role of circRNA in CRC. However, a large number of circRNA remains obscure.

The exosome is a class of extracellular vesicles with a diameter of 30-150nm [8]. It originates from the endolysosomal system and can be secreted into the extracellular fluid. Cell-to cell communication can be achieved by molecules (RNA, proteins, and metabolites), which are abundantly present in exosomes [9]. The function of recipient cells could be modulated when the exosomes were incorporated. Recently, emerging researches demonstrated that exosome-delivered circRNAs promote CRC migration [10] and chemotherapy [11]. However, researches on exosome-delivered circRNAs and their function in CRC are rare.

In this study, we examined the function and mechanism of circCOG2 in CRC. We used the bioinformatics analysis of the public database and found that circCOG2 was upregulated in CRC. Further experiments confirmed that circCOG2 promotes CRC proliferation and migration through the miR-1305/TGF-β2/SMAD3 pathway. Moreover, we found that exosome-delivered circCOG2 could be transmitted among the subpopulations and promote CRC progression.

## Results

### circCOG2 is upregulated in CRC tissues and is associated with poor prognosis

**Fig. 1:**
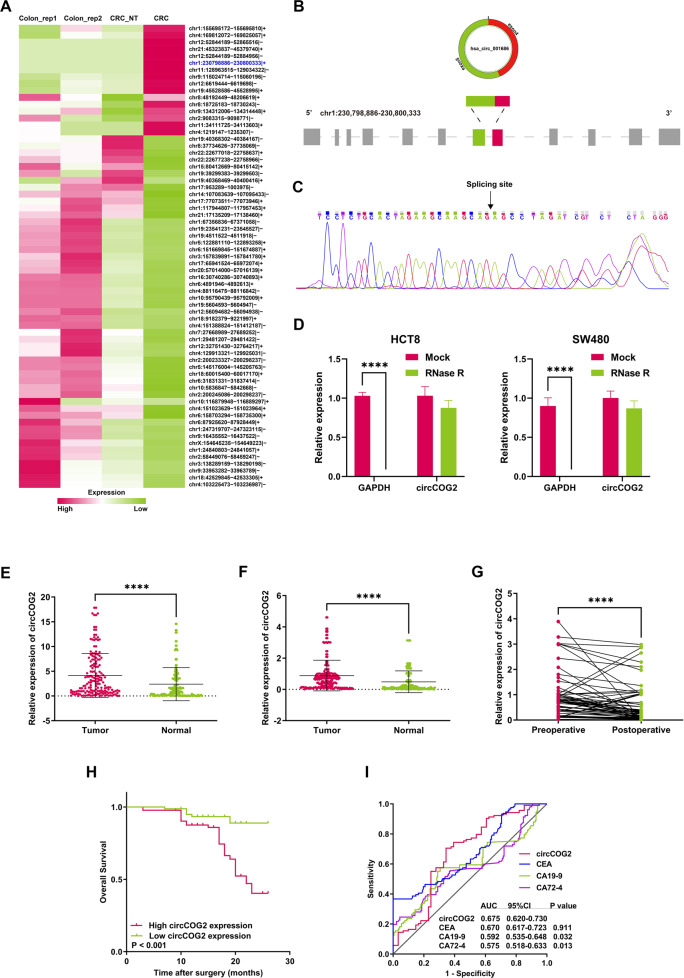
Upregulated circCOG2 in CRC is correlated with poor prognosis. **A** Dysregulated circRNAs in CRC tissues compared with normal tissues (CRC: colorectal cancer; CRC_NT: adjacent non-tumor tissues; CRC_rep: normal mucosa tissues). **B** The gene loci of COG2 and exon 4 and 5 formed circCOG2. **C** Sanger sequence showing the splicing site of circCOG2. **D** The expression level of circCOG2 and GAPDH after RNase R treatment. **E** Expression of circCOG2 in tumor and normal tissues. **F** Expression of circCOG2 in plasma isolated from CRC patients and healthy people. **G** Expression of circCOG2 in preoperative and postoperative plasma of CRC patients. **H** Survival curve of CRC patients with high circCOG2 expression and low circCOG2 expression. **I** The AUC of circCOG2, CEA, CA19-9, and CA 72-4. ****P < 0.0001; *P*-values were calculated by Student’s *t*-test.

To obtain the molecules involved in CRC progression, we analyzed the public database (GSE77661) as previously described [12]. Sixty-seven dysregulated circRNAs with |logFC | > 1 and P < 0.05 were confirmed (Fig. 1A). Among them, circMBOAT2 (chr2:9083315-9098771) and circ0026416 (chr12:52844189-52865516) have been demonstrated to promote CRC progression in our previous research [12, 13]. In this study, we focused on hsa_circ_0016866, which has not been studied so far with the fold change of 2.58 (Tumor vs. Normal). According to Circbase (http://circrna.org/), the gene symbol of hsa_circ_0016866 (named circCOG2) was COG2 (a component of oligomeric Golgi complex 2) and located on chr1:230798886-230800333 (Fig. 1B). Sanger sequence (Fig. 1C) and RNase resistant assay (Fig. 1D) confirmed the circular structure of circCOG2.

In order to explore whether circCOG2 participated in the progression of CRC, we firstly detected the expression level of circCOG2 in CRC tissues. Consistent with the results of sequencing, the expression level of circCOG2 in CRC tissues was significantly higher than that in adjacent non-tumor tissues (Fig. 1E). The CRC patients were then divided into two groups (circCOG2 high/low expression) according to the median value of circCOG2 expression. We found that the expression level of circCOG2 was related to tumor metastasis, pTNM stage, and perineural invasion (Table 1). In addition, the expression of circCOG2 in plasma was higher in CRC patients than in healthy people (Fig. 1F), High expression of circCOG2 resulted as an independent prognostic factor for CRC (Table 2). CRC patients with high circCOG2 expression had lower OS than those with low circCOG2 expression (Fig. 1G). High expression of circCOG2 was an independent prognostic factors for CRC (Table 2). CRC patients with high circCOG2 expression had lower OS than that with low circCOG2 expression (Fig. 1H). The AUC (area of under area under the curve) of circCOG2, CEA, CA19-9, and CA72-4 was 0.675, 0.670, 0.592, and 0.575, respectively (Fig. 1I). These results indicated that circCOG2 is involved in CRC progression and is correlated with a poor prognosis for CRC.

**Table 1:** CircCOG2 expression levels with clinicopathological features derived from CRC patient samples.

Factors	Cases	CircCOG2 expression		*P*-value
	(*n* = 169)	High (*n* = 90)	Low (*n* = 79	
Gender				0.210
Male	116	58	58	
Female	53	32	21	
Age (year)				0.899
≤60	74	39	35	
>60	95	51	44	
Tumor location				0.389
Colon	67	33	34	
Rectum	102	57	45	
Tumor diameter (cm)				0.659
≤5	112	61	51	
>5	57	29	28	
Tumor differentiation				0.418
Poor	34	16	18	
Well / Moderate	135	74	61	
pT stage				0.386
T1–T3	123	63	60	
T4	46	27	19	
pN stage				0.883
N0	93	50	43	
N1–N2	76	40	36	
Distant metastasis				**0.047**
No	155	79	76	
Yes	14	11	3	
pTNM stage				**0.032**
I–II	73	32	41	
III–IV	96	58	38	
Lymphovascular invasion				0.954
Yes	19	10	9	
No	150	80	70	
Perineural invasion				**0.007**
Yes	34	8	0	
No	135	82	79	

All *P* values < 0.05 were marked in bold print.

**Table 2:** Univariate and multivariate analysis of clinicopathologic factors for overall survival.

Factors	Univariate analysis		Multivariate analysis	
	HR (95% CI)	*P*-value	HR (95% CI)	*P*-value
Gender (male vs female)	0.44 (0.17–1.13)	0.088		
Age (≤65 years vs >65 years)	0.85 (0.43–1.68)	0.647		
Tumor diameter (≤5 cm vs >5 cm)	1.96 (0.99–3.85)	0.052		
Tumor location (colon vs rectum)	1.50 (0.76–2.96)	0.240		
Differentiation (well / moderate vs poor)	0.89 (0.39–2.06)	0.794		
pT stage (T1–T3 vs T4)	2.02 (1.02–4.01)	**0.043**	2.23 (1.06–4.67)	**0.035**
pN stage (N0 vs N1–N2)	1.60 (0.80–3.20)	0.188		
Distant metastasis (M0 vs M1)	7.11 (3.37–15.00)	**<0.001**	5.66 (2.5–12.83)	**<0.001**
pTNM stage (I–II vs III–IV)	5.28 (2.15–13.01)	**<0.001**	3.49 (1.38–8.8)	**0.008**
CircCOG2 expression (low vs high)	3.97 (1.64–9.61)	**0.002**	2.70 (1.08–6.72)	**0.033**

*HR* hazard ratio, *CI* confidence interval.

All *P* values < 0.05 were marked in bold print.

### circCOG2 promotes CRC cells proliferation, migration, and invasion

**Fig. 2:**
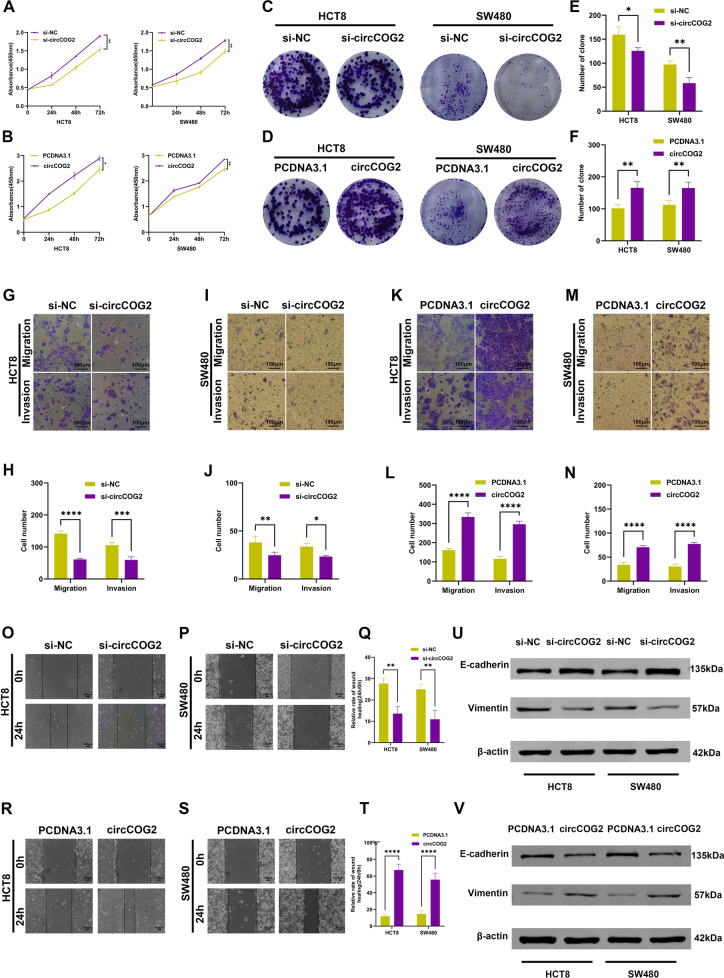
circCOG2 promotes CRC proliferation, migration, and invasion in vitro. **A**, **C** and **E** CCK8 and colony formation assays indicated that knockdown of circCOG2 inhibited CRC cells proliferation. **B**, **D** and **F** CCK8 and colony formation assays indicated that overexpression of circCOG2 promoted CRC cells proliferation. **G**–**J** Transwell assay indicated that knockdown of circCOG2 inhibited CRC cells migration and invasion. **K**–**N** Transwell assay indicated that overexpression of circCOG2 promoted CRC cells migration and invasion. **O**–**Q** Wound healing assay indicated that knockdown of circCOG2 inhibited CRC cells migration. **R**–**T** Wound healing assay indicated that overexpression of circCOG2 promoted CRC cells migration. **U** Western blot showed that the expression of vimentin and E-cadherin was decreased and increased, respectively, in CRC cells with circCOG2 knockdown. **V** Western blot showed that the expression of vimentin and E-cadherin was increased and decreased, respectively, in CRC cells with circCOG2 overexpression. *P < 0.05; **P < 0.01; ***P < 0.001; ****P < 0.0001; *P*-values were calculated by Student’s *t*-test.

The expression of circCOG2 in CRC cells was higher than that in the normal intestinal epithelial cell line (HCO) (Supplementary Fig. S1A). The HCT8 and SW480 cells were chosen for further study. To investigate the function of circCOG2 in CRC progression, we designed three siRNAs to knock down the circCOG2 expression. The expression level of circCOG2 was significantly inhibited in these two cell lines by si-1 (referred to as si-circCOG2 hereafter) (Supplementary Fig. S1B). Moreover, the CCK-8 assay and colony formation assay demonstrated that knockdown of circCOG2 inhibited cell proliferation (Fig. 2A, C, E). Trans-well and wound healing assays revealed that circCOG2 knockdown could inhibit cell invasion and migration viability. With the upregulated E-cadherin and downregulated vimentin (Fig. 2G, H, I, J, O, P, and Q), Western blot showed that circCOG2 knockdown decreased the expression of vimentin and increased the expression of E-cadherin (Fig. 2U).

Next, we cloned circCOG2 into the PCDNA3.1 plasmid and transfected HCT8 and SW480 cells with PCDNA3.1-circCOG2 or PCDNA3.1. The overexpression efficiency was detected 48 h later (Supplementary Fig. S1C). It was revealed that overexpression of circCOG2 significantly promoted cell proliferation, invasion, and migration (Fig. 2B, D, F, K, L, M, N, R, S, T). Also, Western blot showed that circCOG2 promoted vimentin expression and inhibited E-cadherin expression (Fig. 2V).

### miR-1305 is the target of circCOG2

**Fig. 3:**
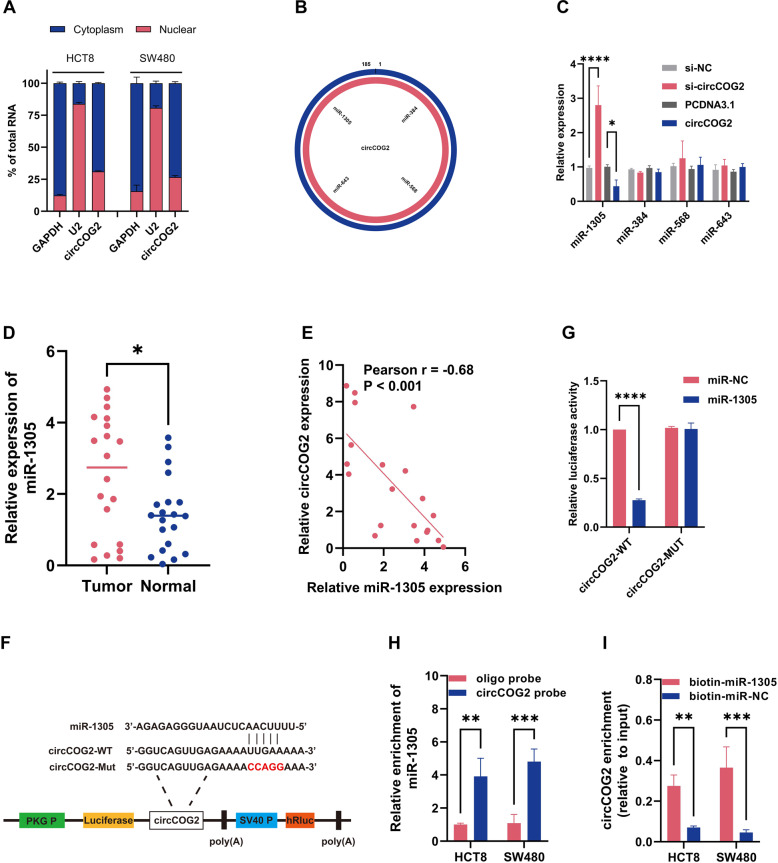
miR-1305 is the target of circCOG2. **A** Cytoplasm and nuclear fractions indicated that circCOG2 was located in the cytoplasm. **B** The illustration showed the binding site of four miRNAs with circCOG2. **C** The expression of four miRNAs in circCOG2 knockdown or overexpression cells. **D** The expression of miR-1305 in CRC and normal tissues. **E** The expression of miR-1305 was negatively correlated with the expression of circCOG2. **F** The illustration of the dual-luciferase reporter plasmid. **G** The luciferase activity of circCOG2-WT or circCOG2-MUT transfected with miR-1305 or miR-NC. **H** CircCOG2 probe pull more miR-1305 than oligo probe. **I** MiR-1305 pulled more circCOG2 than miR-NC. *P < 0.05; **P < 0.01; ****P < 0.0001; *P*-values were calculated by Student’s *t*-test.

We found that circCOG2 was mainly located in the cytoplasm (Fig. 3A and Supplementary Fig. S1J). Previous studies revealed that circRNAs in the cytoplasm usually acted as competitive RNA (ceRNA) for miRNA to promote CRC progression [5, 6]. Therefore, we speculated that circCOG2 could compete with certain miRNAs. By CircInteractome (https://circinteractome.irp.nia.nih.gov/index.html), four miRNAs (miR-1305, miR-384, miR-568, and miR-643) were predicted to be the target of circCOG2 (Fig. 3B). We detected the expression of these four miRNAs in CRC cells. Only miR-1305 was downregulated in circCOG2-overexpression cells and upregulated in circCOG2-knockdown cells (Fig. 3C). Next, we detected the expression of miR-1305 in CRC tissues, which revealed that the expression of miR-1305 was higher in CRC tissues compared to adjacent non-tumor tissues (Fig. 3D). Moreover, the expression of miR-1305 was negatively correlated with circCOG2 (Fig. 3E). We cloned the binging sequence of circCOG2 (circCOG2-WT) or mutant (circCOG2-MUT) to the dual-luciferase reporter plasmid (Fig. 3F). The luciferase activity was significantly reduced when the cell was transfected with the circCOG2-wild type (circCOG2-WT) and miR-1305 mimics. However, the luciferase activity did not change when the cell was transfected with the circ-mutant type (circCOG2-Mut) and miR-1305 or miR-NC (Fig. 3G). The biotin-labeled RNA pull-down assay further confirmed that circCOG2 could couple with miR-1305 (Fig. 3H, I). The above results demonstrated that miR-1305 is the target of circCOG2.

### miR-1305 inhibits CRC progression and weakens the pro-tumor effect of circCOG2

**Fig. 4:**
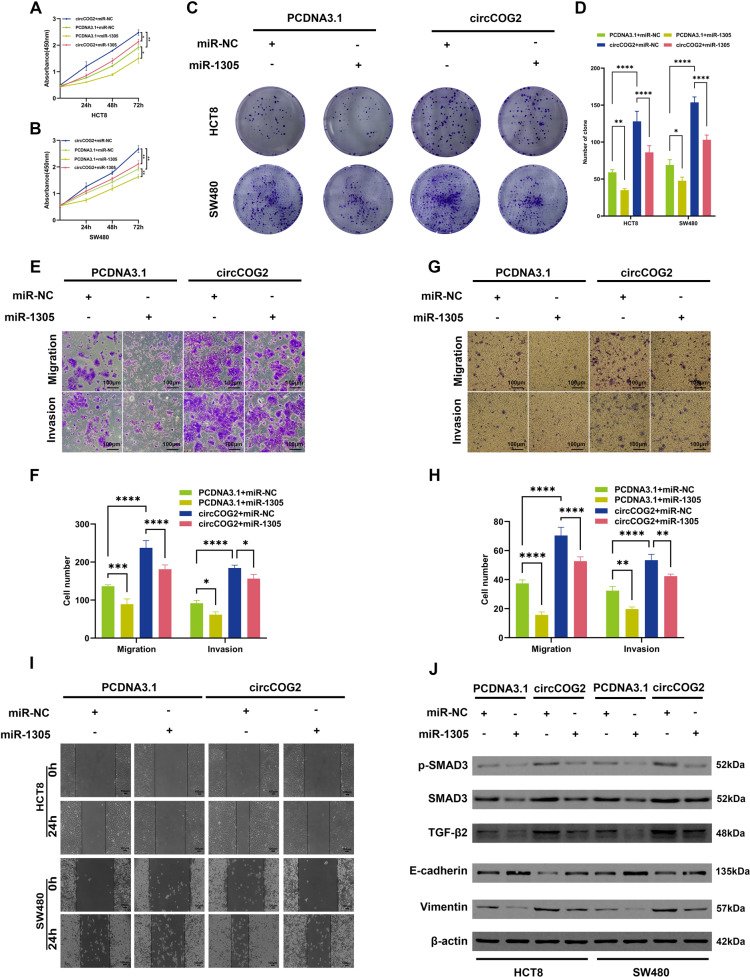
miR-1305 inhibits CRC proliferation, migration, and invasion. **A**–**D** CCK-8 and colony formation assays indicated that miR-1305 inhibited CRC proliferation and rescued the pro-proliferation effect of circCOG2. **E**–**H** Transwell assay indicated that miR-1305 inhibited CRC cells migration and invasion and rescued the pro-metastasis effect of circCOG2. **I** Wound healing assay indicated that miR-1305 inhibited CRC cells migration and rescued the pro-migration effect of circCOG2. **J** Western blot showed that miR-1305 upregulated the expression of E-cadherin and downregulated the expression of vimentin, TGF-β2, SMAD3, and p-SMAD3. Mir-1305 rescued the pro-EMT and TGF-β2/SMAD3 pathway activation effect of circCOG2. *P < 0.05; **P < 0.01; ****P < 0.000; P values were calculated by Student’s t-test.

The transfection efficiency of miR-1305 mimics was quantified by qRT-PCR (Supplementary Fig. S1D). Compared with cells transfected with miR-NC, the proliferation, migration, and invasion were significantly inhibited by miR-1305 (Fig. 4A–I). However, the pro-proliferation, pro-migration, and pro-invasion effect of circCOG2 could be rescued by miR-1305 (Fig. 4A–I). E-cadherin was downregulated, and vimentin was upregulated when circCOG2 was overexpressed (Fig. 4J), and this effect was rescued by miR-1305 mimics (Fig. 4J). Taken together, these results indicated that circCOG2 promotes CRC progression by sponging miR-1305.

### circCOG2 promotes CRC EMT by miR-1305/TGF-β2/SMAD3 pathway

**Fig. 5:**
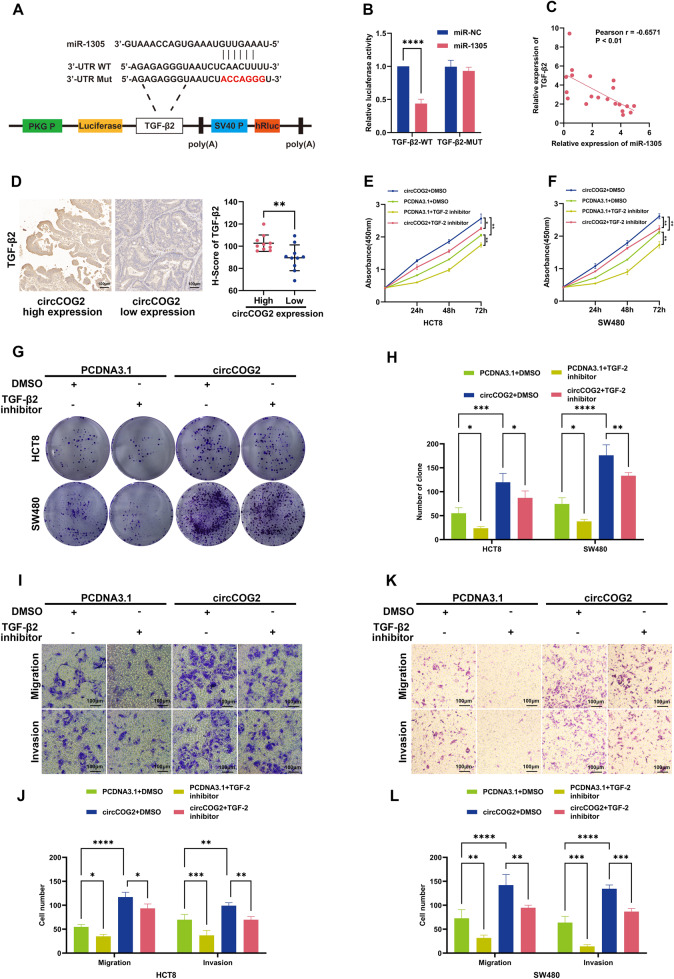
CircCOG2 promotes CRC proliferation, migration, and invasion via the miR1305/TGF-β2 pathway. **A** The illustration of dual luciferase reporter plasmid. **B** The luciferase activity of TGF-β2-WT or TGF-β2-MUT transfected with miR-1305 or miR-NC. **C** The expression of TGF-β2 was negatively correlated with miR-1305. **D** Represent imagines for IHC of TGF-β2 in circCOG2 high/low expression group and their IHC score. **E**–**H** CCK-8 and colony assays indicated that the TGF-β2 inhibitor rescued the pro-proliferation effect of circCOG2. **I**–**L** Transwell assay indicated that TGF-β2 inhibitor rescued the pro-invasion and pro-migration effect of circCOG2. *P < 0.05; **P < 0.01; ***P < 0.001; ****P < 0.0001; *P*-values were calculated by Student’s *t*-test.

TGF-β2 was the target of miR-1305 predicted by miRBD (http://mirdb.org/) and Targetscan (http://www.targetscan.org/mamm_31/). A previous study demonstrated that miR-1305 inhibited bladder cancer progression by the TGF-β2/SMAD3 pathway [14]. By analyzing 41 paired of CRC tissues from the TCGA database (https://www.tnmplot.com/), we found that the gene expression of TGF-β2 was higher in tumors than in normal tissues (Supplementary Fig. S1E). The dual-luciferase reporter plasmid, including the binding (TGF-β2-WT) or mutant sequence (TGF-β2-MUT), was constructed (Fig. 5A). Compared with cells transfected with TGF-β2-WT plasmid and miR-NC, the luciferase activity was significantly reduced when the cell was transfected with TGF-β2-WT plasmid and miR-1305 mimics. However, no change was detected when cells were transfected with TGF-β2-MUT and miR-1305 mimics or miR-NC (Fig. 5B). qRT-PCR demonstrated that the expression of TGF-β2 was negatively correlated with miR-1305 (Fig. 5C).

Next, we used IHC for TGF-β2 in 20 CRC tissues and divided them into two groups according to the median value of the expression of circCOG2. The IHC score of the circCOG2 high expression group was significantly higher than that in the circCOG2 low expression group (Fig. 5D). Western blot indicated that miR-1305 reduced the expression of TGF-β2, vimentin, SMAD3, and p-SMAD3, and increased the expression of E-cadherin (Fig. 4J). We concurrently transfected cells with circCOG2-overexpression plasmid and miR1305 mimics or miR-NC and found that the pro-EMT and TGF-β2 activation effect by circCOG2 was rescued by miR-1305 with upregulated E-cadherin and downregulated vimentin, TGF-β2, SMAD3, and p-SMAD3 (Fig. 4J). CircCOG2 promoted cell proliferation, migration, and invasion, but these effects were rescued by TGF-β inhibitor (Fig. 5E–L). These results demonstrated that circCOG2 promotes CRC EMT by miR-1305/TGF-β2/SMAD3 pathway.

### Exosome-transmitted circCOG2 promotes CRC progression

**Fig. 6:**
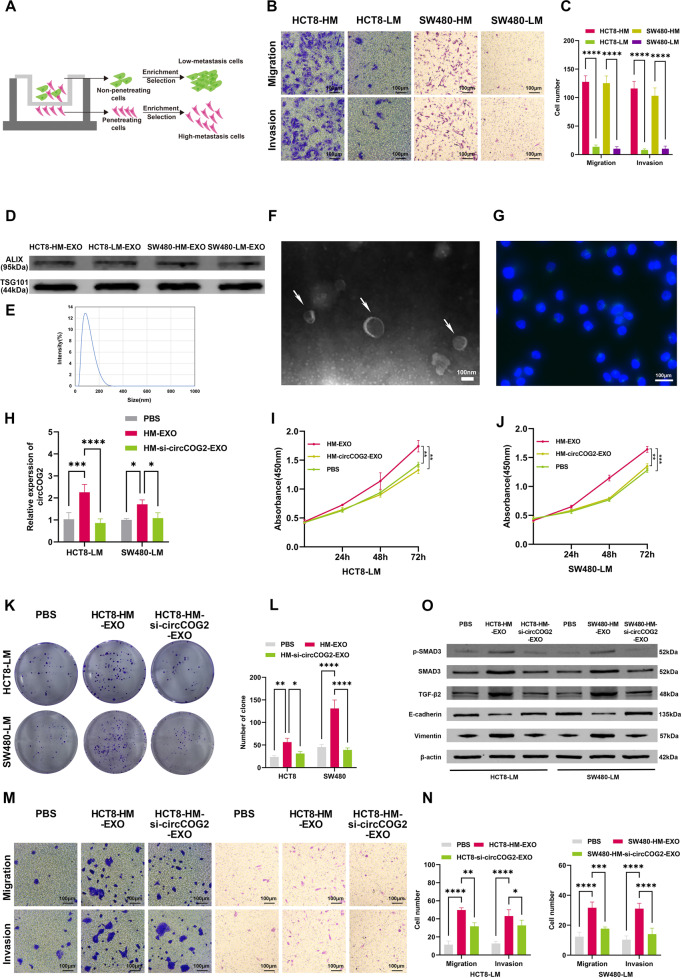
Exo-transmitted circCOG2 promotes CRC progression in vitro. **A** The illustration of establishing CRC cell lines with high metastatic potential or low metastatic potential. **B**, **C** The metastatic potential of CRC cells with different metastatic potential was confirmed by transwell assay. **D** Western blot showed the marker of exosomes. **E** NTA showed the size of exosomes. **F** TEM showed the microstructure of exosomes (white arrows). **G** The exosomes were stained by PKH67 (green fluorescence) were cocultured with CRC cells, and cell nuclear were stained by DAPI (blue fluorescence). **H** The expression of circCOG2 in cell cocultured with PBS, HM-EXO or HM-si-circCOG2-EXO. **I**–**L** CCK-8 and colony formation assays indicated exo-transmitted circCOG2 promoted the proliferation of CRC cells with low metastatic potential. **M**, **N** Transwell assay indicated that exo-transmitted circCOG2 promoted migration and invasion of CRC cells with low metastatic potential. **O** Exosome-transmitted circCOG2 promoted EMT and TGF-β2/SMAD3 pathway. *P < 0.05; **P < 0.01; ***P < 0.001; ****P < 0.0001; *P*-values were calculated by Student’s t-test.

Previous studies revealed that some non-coding RNAs could be transmitted among cells through exosomes, which in turn affected the characteristic of recipient cells [15, 16]. To further explore this, we established two CRC cell lines with high metastatic potential (named as HCT8-HM and SW480-HM) and two CRC cell lines with low metastatic potential (named as HCT8-LM and SW480-LM) as previously described [17] (Fig. 6A). Metastatic and invasive potential were detected by trans-well assay (Fig. 6B, C). qRT-PCR showed that the expression of circCOG2 was higher in HCT8-HM and SW480-HM cells compared to HCT8-LM and SW480-LM cells (Supplementary Fig. S1F). Exosomes were isolated and identified by Western bolt (Fig. 6D), NTA (Fig. 6E) and TEM (Fig. 6F). The expression of circCOG2 was higher in exosomes isolated from HCT8-HM (HCT8-HM-EXO) and SW480-HM (SW480-HM-EXO) cells than in exosomes isolated from HCT8-LM (HCT8-LM-EXO) and SW480-LM cells (HCT8-LM-EXO)(Supplementary Fig. S1G). Then, we transfected HCT8-HM and SW480-HM cells with si-circCOG2 and isolated the exosomes from these two cell lines (HCT8-HM-si-circCOG2-EXO and SW480-HM-si-circCOG2-EXO). qRT-PCR revealed that the expression of circCOG2 in exosomes could be knocked down by si-circCOG2 (Supplementary Fig. S1H). The exosomes were labeled with PKH67 and cocultured with recipient cells. The green fluorescence could be observed around the nucleus of the recipient cells (Fig. 6G). We found that the expression of circCOG2 increased when recipient cells were cocultured with EXO-HM for 48 h, but this effect was not observed by HM-si-circCOG2-EXO (Fig. 6H). The exosome-transmitted circCOG2 promoted the proliferation and migration viability of HCT8-LM and SW480-LM cells, and this effect could be rescued by si-circCOG2 (Fig. 6I–N). Also, the exosome-transmitted circCOG2 elevated the expression of vimentin, TGF-β2, SMAD3, p-SMAD3, and decreased the expression of E-cadherin, which could be recuperated by si-circCOG2 (Fig. 6O). Our results demonstrated that exosome-circCOG2 could be transmitted from CRC cells with the high metastatic potential to CRC cells with low metastatic potential and that exosome-circCOG2 promotes the proliferation, invasion and migration of recipient cells.

### Exosome-transmitted circCOG2 promotes CRC progression in vivo

**Fig. 7:**
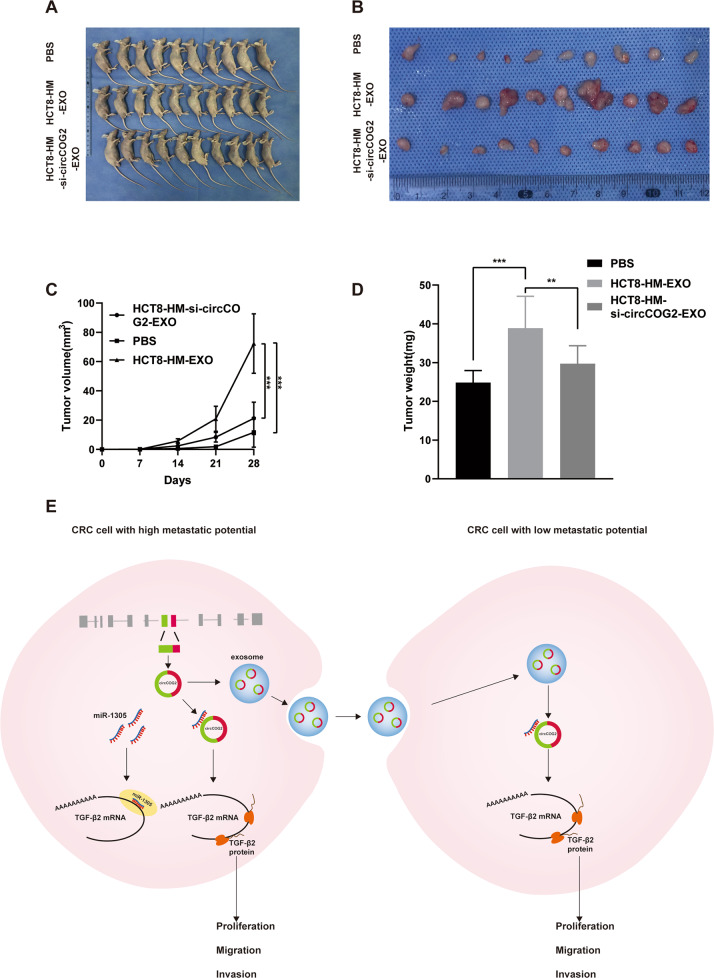
Exo-transmitted circCOG2 promotes CRC progression in vivo. **A** The xenograft tumors assay showed the tumors after four weeks of growth. **B** The represented image of the removal tumor. **C** The volume of the tumor measured once a week in each group. **D** The final weight of tumor in each group. **E** The molecular mechanism of circCOG2 in CRC. *P < 0.05; **P < 0.01; *P* values were calculated by Student’s *t*-test.

HCT8-LM cells were used for xenograft tumor models to investigate the role of exosome-transmitted circCOG2 in CRC. The first group was injected with 10 μg of HCT8-HM-EXO every two days. The second group was injected at the tumor site with equal PBS. The last group was injected with 10 μg of HCT8-HM-si-circCOG2-EXO. The volume and weight of the first group were significantly higher than that of the second group. However, with the decrease of circCOG2 (Supplementary Fig. S1I), the growth of the tumor was significantly slowed down (Fig. 7A–D). Taken together, the above results demonstrated that exosome-transmitted circCOG2 promotes CRC progression in vitro and in vivo (Fig. 7E).

## Discussion

circRNA, first discovered in humans in 1993, was initially considered as the product of mis-splicing [18]. Due to recent advancements in sequence technology, many circRNAs were found in human tissues, whereas dysregulated circRNAs were related to diseases such as CRC. For example, hsa_circ_101555 is increased in CRC, while upregulated hsa_circ_101555 promotes cell proliferation and inhibits apoptosis [5]. Higher expression of hsa_circ_001680 was found in CRC compared to normal tissues, and hsa_circ_001680 promoted CRC proliferation, migration, and chemoresistance [6]. Our results revealed that circCOG2 was abnormally upregulated in CRC tissues and plasma. The expression of circCOG2 was correlated with clinicopathological factors, including distant metastasis, pTNM stage, and perineural invasion. Up-regulation or downregulation of circCOG2 promoted or inhibited CRC proliferation, migration, and invasion, respectively. These results indicated that circCOG2 had an important role in CRC progression, which urged us to study the mechanism further.

A few circRNAs are retained in the nucleus where they regulate transcription and splicing. E.g., downregulates circSMARCA5 in nuclear-induced chemoresistance of breast cancer by inhibiting the transcription of SMARCA5 [19]. Moreover, circLONP2 promotes CRC migration and invasion by regulating the maturation of pri-miR-17 [20]. Yet, most circRNAs are located in the cytoplasm and act as a miRNA sponge [2]. Hsa_circ_101555 sponge miR-597-5p to increase the expression of cyclin-dependent kinase 6 and replication protein A3 [5]. Hsa_circ_001680 upregulates the expression of B cell-specific Moloney murine leukemia virus integration site 1 by sponging miR-340 [6].

In this study, we firstly detected the location of circCOG2 and found circCOG2 was mainly located in the cytoplasm. We speculated that circCOG2 promotes CRC progression by sponging certain miRNA. Four miRNAs were predicted as the targets of circCOG2. Only the expression of miR-1305 changed with the upregulation or downregulation of circCOG2. The expression of circCOG2 was negatively associated with miR-1305. The dual-luciferase reporter assay revealed the binging site between circCOG2 and miR-1305. The biotin-labeled RNA pull-down assay proved the direct combination of circCOG2 and miR-1305. In addition, a series of rescued experiments demonstrated that circCOG2 promoted CRC proliferation and migration by sponging miR-1305.

Tumor metastasis has been identified as a risk factor for the death of CRC, with the 5-year OS for metastatic CRC being only 14% [1]. Epithelial-to-mesenchymal transition (EMT) is an important mechanism for tumor metastasis, where TGF-β has been identified as the most potent inducer of EMT [21, 22]. The combination of ligand and receptor activates the SMAD protein, and phosphorylated SMAD enters the cell nucleus and regulates the gene expression [22]. Much evidence has confirmed the role of the TGF-β/SMAD pathway in promoting tumor metastasis. Tang et al. [23] found that the expression of SIRT7 is lower in breast cancer lung metastases, and downregulated SIRT7 promotes EMT of breast cancer via activating TGF-β signal and SMAD4 accumulation. In the present study, we found that circCOG2 was closely correlated with distant metastasis, pTNM stage, and perineural invasion, and high expression of circCOG2 predicted poor prognosis, which indicated that circCOG2 is involved in CRC metastasis. Function assays further revealed that circCOG2 promotes CRC proliferation, migration, and invasion. Mechanistically, circCOG2 sponge miR-1305 to activate the TGF-β2/SMAD3 pathway. In a previous study, Luo et al. [24] demonstrated that lncRNA CASC9 promotes CRC growth and inhibits apoptosis by regulating the TGF-β2/SMAD3 pathway. Sun et al. revealed that circRIP2 promotes bladder cancer metastasis through the miR-1305/TGF-β2/SMAD3 pathway [14]. T These results highlight the important role of the TGF-β2/SMAD3 pathway in cancer progression.

Numerous studies have demonstrated that exosome-transmitted molecules promote CRC progression [10, 11, 20]. Considering the heterogeneity, molecules transferred by exosomes are involved in intercellular communication and change the characteristic of sublines in a single tumor. Qu et al. [15] established that the sunitinib-resistant renal cell carcinoma cells and exosome-transmitted lncARSR from sunitinib-resistant cells disseminated sunitinib resistance. Han et al. [20] d demonstrated that miR-17 could be transferred from CRC cells with the high metastatic potential to cells with low metastatic potential by exosomes, thus disseminating CRC metastasis. In order to investigate whether circCOG2 was involved in intercellular communication in CRC cell sublines via exosomes, we established CRC cells with different metastatic potential. Our results revealed that CircCOG2 was regulated in CRC cells with high metastatic potential and their secreted exosomes. The recipient cells, CRC cells with low metastatic potential, enhanced the proliferation, migration, and invasion when cocultured with exosomes extracted with CRC cells with high metastatic potential. This effect was not observed when the cells were cocultured with cells transfected with si-circCOG2. Our results demonstrated that exosome-transmitted circCOG2 promoted CRC progression.

In conclusion, high expression of circCOG2 was closely correlated to the advanced stage and predicted poor prognosis. CircCOG2 was located in the cytoplasm and acted as a sponge for miR-1305 to activate the TGF-β2/SMAD3 pathway. Exosome-transmitted circCOG2 promoted CRC progression in vivo and in vitro. Our research provides a promising target for CRC.

## Materials and methods

### Patients and samples

From April 2012 to September 2014, 212 CRC patients who underwent surgical resection in Qilu Hospital (Jinan, China) were enrolled in this study. During the same period, 183 healthy individuals were recruited as controls. Patients did not receive any pre-surgical treatment such as chemotherapy, radiotherapy or targeted therapy. In total, 169 paired CRC tissues and adjacent normal tissues, 212 pre-surgical plasma samples, and 113 paired pre- and post-surgical plasma samples were collected from CRC patients. In addition, 183 plasma samples were obtained from healthy individuals. The cancerous tissues and its adjacent normal tissues as well as plasma samples were immediately stored in liquid nitrogen. This study was approved by the Ethics Committee of Qilu Hospital, Shandong University (Jinan, China). Each study participant provided informed consent.

### RNA preparation and quantitative real-time PCR (qRT-PCR)

TRIzol and TRIzol LS reagent (Thermo Fisher Scientific, USA) were used to extract the total RNA from the tissues and plasmas, respectively. Nuclear and cytoplasmic fractionations were extracted using PARIS Kit (Invitrogen, USA). The RNA was reversely transcribed to cDNA by using PrimeScriptTM RT Reagent Kit (Takara, Japan). GAPDH, U2 and U6 were used as the reference for cytoplasmic, nuclear fractions and miRNA, respectively. The relative expression level was calculated using the 2^-ΔΔCT^ method. The primers were listed in Supplementary Table. S1.

### RNase R treatment

2 μg of total RNA was treated with 6U of RNase R (Epicentre Biotechnologies, USA) and incubated for 20 min at 37 °C. Then the RNA was reversely transcribed into cDNA, and the expression level of circCOG2 and GAPDH were detected by qRT-PCR.

### Cell culture

Normal intestinal epithelial cell line (HCO) and DLD1, SW480 and HCT8 cell lines were presented by pathology laboratory of Shandong university. The trans-well chambers (8μm, coring, USA) was used to establishing the CRC cell lines with high metastatic potential or low metastatic potential as previous described [17]. All cells were maintained in Dulbecco’s modified eagle medium (DMEM, Gibco, USA) supplemented with 10% fetal bovine serum (FBS) and incubated at 37 °C and 5% CO_2_.

### Cell transfection

Short interfering RNA (siRNA), PCDNA3.1-circCOG2 plasmid and corresponding negative control (si-NC, PCDNA3.1) were designed and synthesized by Hanbio Biotechnology (Shanghai, China). Micro RNA (miRNA) mimics were designed and synthesized by GenePharma Biotechnology (Shanghai, China). The cells were seeded into plates according to the manufacturer’s instructions. When the cell density reached 60%, the transfection was started with Lipofectamine 3000 (ThermoFisher, USA). 48 h later, the cells were collected and used for the further experiments.

### Cell counting Kit-8 (CCK-8) assay

3×10^3^ cells/well were seeded in 96-well plates and cultured for 0, 24, 48, 96 h, then CCK-8 (APExBIO Technology, USA) at 10 μL/well was added. Two hours later, cell viability was detected at the optical density (OD) with 450 nm by a microplate reader (ThermoFisher, USA).

### Colony formation assay

600 cells/well were plated in 6-well plates. Change the medium every two weeks. Two weeks later, cell colonies were fixed with methanol and stained with crystal violet.

### Trans-well assay

The trans-well plates (8μm, Corning, USA) were used to measure the migrative and invasive viability of the CRC cells. For migration assay, 600 μL medium containing 20% FBS was added to the lower chamber, and 2×10^5^ cells in 200 μL medium without FBS were plated into the upper chamber. For invasion assays, 6 h prior to the experiment, matrixgel (Corning, USA) was placed in the upper chamber, with additional steps similar to those of the migration assay. 24 h later, cells on the upper membrane were removed, and cells in the lower membrane were fixed with methanol and stained with crystal violet.

### Wound healing assay

2×10^5^ cells/well were seeded in the 6-well plate. The scratch was made by a 200 μl pipette tip When cell density reached 100%. The cells were washed with PBS, and the medium was replaced with FBS-free medium. 24 h later, the cells were washed three times with PBS, and the healing area was calculated by Imagine-Pro Plus 6.0 (Media Cybernetics, USA).

### Dual-luciferase reporter assay

The pmirGLO plasmid with circCOG2-WT, circCOG2-MUT, TGFβ2-WT or TGFβ2-MUT were designed and synthesized by Hanbio Biotechnology (Shanghai, China). The luciferase activities were detected by Dual-Luciferase Reported Assay System (Promega, USA). The details were described in our previous research [13].

### Biotin-labeled RNA pull-down

The procedures were performed as previous described [25]. In brief, biotin-labeled circCOG2 or oligo probe were designed synthesized by GenePharma Biotechnology (Shanghai, China). Streptavidin-coated magnetic beads were added into the probe and incubated for 2 h. The lysate of 1 × 10^7^ cells was incubated with the probe-coupled magnetic beads at 4 °C for 24 h. The coupled miRNAs were eluted, extracted, and detected by qRT-PCR.

For the biotin-labeled miRNA pull down assay, the cells were transfected with PCDNA3.1-circCOG2 plasmid. Then the cells were transfected with biotin-labeled miR-1305 or miR-NC, and harvested 48 h later. The lysis buffer was incubated with magnetic beads and blocked with yeast tRNA. The coupled RNA was extracted from the cell lysates and detected by qRT-PCR.

### Immunohistochemistry staining

The CRC tissues sections were immunohistochemistry (IHC) using TGF-β2 antibodies (1:200; Proteintech). The sections scanner (PANNORAMIC, 3DHISTECH) was used for images acquisition. Quant Center 2.1 software (3DHISTECH)) was used to quantify the IHC score (IHC Score = ∑ (PI × I) = (percentage of cells of weak intensity × 1) + (percentage of cells of moderate intensity × 2) + percentage of cells of strong intensity × 3), pi represented the ratio of positive signal pixel area, I represented the coloring intensity).

### Exosome isolation and identification

Cells were cultured in the 10 cm dish. When the density reached 60%, the cells were washed three times with PBS and the medium was replaced with 10% exosome-free FBS medium. 48 h later, the medium was collected and centrifuged as previous described [11]. The exosomes were resuspended in PBS, filtered with 0.2μm filters and stored at -80 °C.

Exosomes were identified by Transmission Electron Microscopy (TEM), Nanoparticle Tracking Analysis (NTA) and western blot.

### PKH67 staining

5 μl of PKH67 (Sigma, USA) was added into 1 ml exosome isolated from 30 ml medium and incubated for 15 min at room temperature. Added 1 ml of 1% bovine serum albumin to terminated the labeling. After ultracentrifuging, the labeled exosomes were resuspended with 100 μl PBS and cocultured with recipient cells for 3 h at 37 °C. The nucleus was stained with 4′,6-diamidino-2-phenylindole (DAPI). The labeled exosomes were observed by fluorescent microscope.

### Western blot

Protein was extracted with RIPA Lysis Buffer (Beyotime Biotechnology, China) with protease and phosphatase Inhibitors. Western blot was performed according to the standard procedure. The antibodies used in this research were listed as follows: Vimentin (1:1000; CST), E-cadherin (1:1000; CST), SMAD3 (1:1000; CST), p-SMAD3 (1:1000; CST), TFG-β2 (1:500; Proteintech), β-actin (1:2000; Boster), ALIX (1:400; Boster), TSG101 (1:400; Boster).

### Animal experiment

This study was approved by the ethics committee of Qilu Hospital of Shandong University. 5× 10^6^ HCT8-LM cells were subcutaneously injected into the right axil of male BALB/C nude mice. The mice were randomly divided into three groups. The first group were injected with 10 μg exosomes isolated from HCT8-HM cell every two days. The second group were injected at the tumor site with equal PBS. The last group were injected with 10 μg exosomes that isolated from HCT8-HM cells transfected with si-circCOG2. The tumor volume was measured once a week using the formula: Tumor volume (*V*) = 1/2 × length(*L*) × width (*W*)^2^. The mice were sacrificed after 4 weeks, and the tumors were removed and weighted.

### Statistics

Date was analyzed using Prism 9.0 (GraphPad Software, USA). The difference between the two groups were analyzed using students’ t test or ANOVA. The association between clinicopathological factors and circCOG2 expression levels were performed using Chi-squared text. Overall survival analysis with clinicopathologic factors were performed using Cox’s proportional hazards regression model. The receiver operating characteristic (ROC) and Kaplan–Meier analysis was used to e. *P* < 0.05 was considered statistically significant.

## Supplementary information

Figure S1

Fig. S1 legends

Table S1

language certificate

author contribution

## Data Availability

All the data were provided from this article or the corresponding author upon reasonable request.
