# Supplementary figures and images for: Exosome-transmitted circCOG2 promotes colorectal cancer progression via miR-1305/TGF-β2/SMAD3 pathway
Source: Cell Death Discov. 2021 Oct 11;7:281. doi: 10.1038/s41420-021-00680-0 (PMC8505430; doi:10.1038/s41420-021-00680-0)

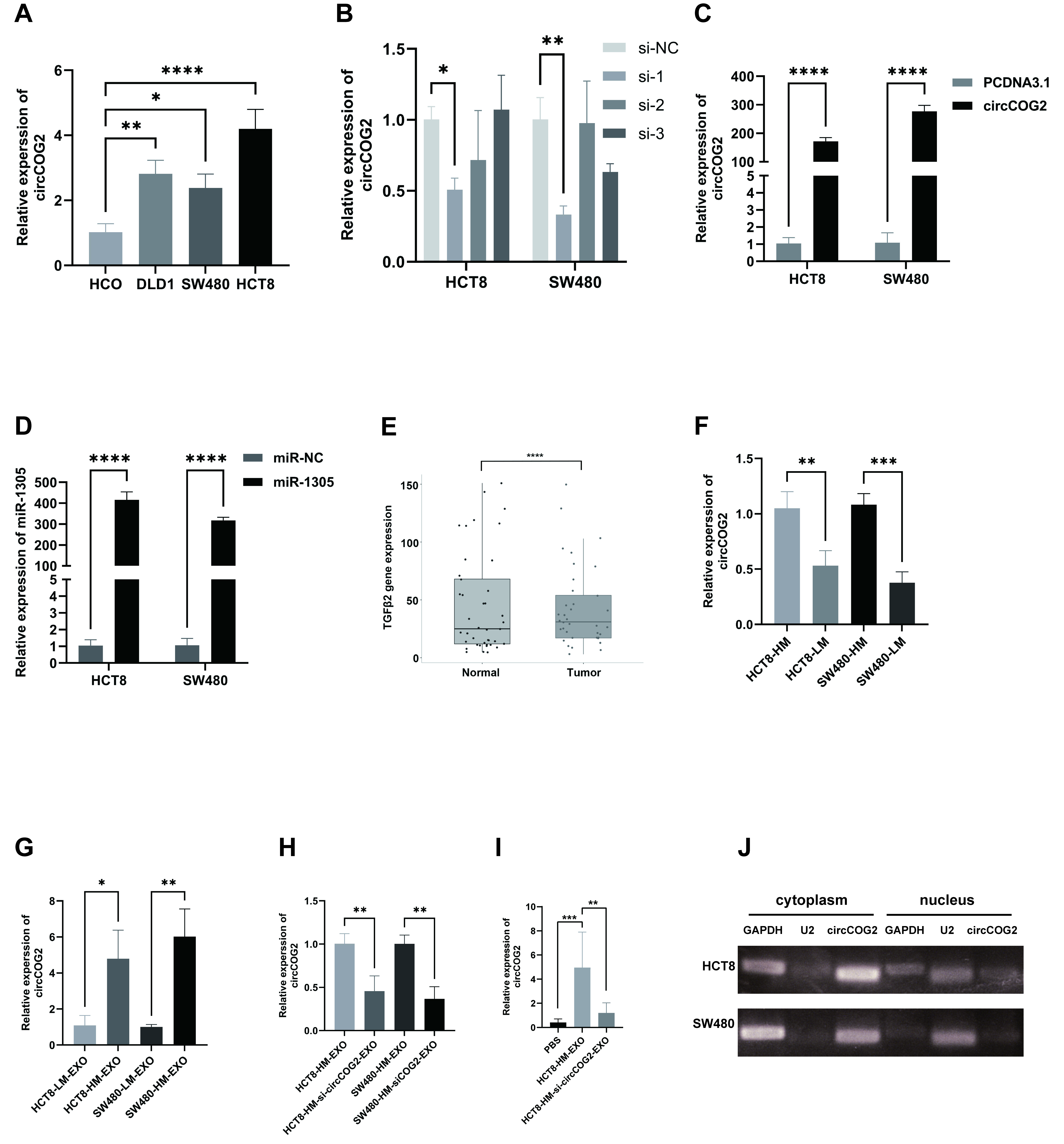

Supplement: Supplementary file 1 — Figure S1 [file 41420_2021_680_MOESM1_ESM.tif]
