# Supplementary material for: Exosome-transmitted circCOG2 promotes colorectal cancer progression via miR-1305/TGF-β2/SMAD3 pathway
Source: Cell Death Discov. 2021 Oct 11;7:281. doi: 10.1038/s41420-021-00680-0 (PMC8505430; doi:10.1038/s41420-021-00680-0)
Supplement: Supplementary file 2 — Fig. S1 legends [file 41420_2021_680_MOESM2_ESM.docx]

**Figure legends**

**Fig. S1** (A) The expression level of circCOG2 in HCO, DLD1, SW480, and HCT8 cell lines. (B) The knockdown efficiency of circCOG2 by siRNAs. (C) The overexpression efficiency of circCOG2 by PCDNA3.1 plasmid. (D) The expression level of miR-1305 after cells transfected with miR-1305 mimics or miR-NC. (E) TGF-β2 gene expression in normal or CRC tissues. (F) The expression level of circCOG2 in CRC cells with high/low metastatic potential. (G) The expression level of circCOG2 in exosomes extracted from CRC cells with high/low metastatic potential. (H) The expression of circCOG2 in exosomes extracted from CRC cells with high metastatic potential or the cells transfected with si-circCOG2. (I) The expression of circCOG2 in the engrafted tumors. (J) The electrophoresis for the RNA separated from the nucleus/cytosol. *P < 0.05; **P< 0.01; *** P < 0.001; ****P < 0.0001; P values were calculated by Student’s t-test.
