# Supplementary material for: Exosome-transmitted circCOG2 promotes colorectal cancer progression via miR-1305/TGF-β2/SMAD3 pathway
Source: Cell Death Discov. 2021 Oct 11;7:281. doi: 10.1038/s41420-021-00680-0 (PMC8505430; doi:10.1038/s41420-021-00680-0)
Supplement: Supplementary file 3 — Table S1 [file 41420_2021_680_MOESM3_ESM.docx]

| **Table S1.** Primers and siRNA sequences | |
| --- | --- |
| Primers used for qRT-PCR |  |
| GAPDH F | GGCCTCCAAGGAGTAAGACC |
| GAPDH R | AGGGGAGATTCAGTGTGGTG |
| CircCOG2 F | TTCGGTCAGTTGAGAAAATTGA |
| CircCOG2 R  TGF-β2 F  TGF-β2 R | ACTGCCCGAATTCCTTCAC  AGAGGGATCTAGGGTGGAAATGGATAC  CCTGCTGTGCTGAGTGTCTGAAC |
| siRNAs oligonucleotides |  |
| NC | UUCUCCGAACGUGUCACGUdTdT |
|  | ACGUGACACGUUCGGAGAAdTdT |
| si-1 | AAGCAGAGCCUUAGAUCGUCUdTdT |
|  | AGACGAUCUAAGGCUCUGCUUdTdT |
| si-2 | ACUAGAAGCAAGCAGAGCCUUdTdT |
|  | AAGGCUCUGCUUGCUUCUAGUdTdT |
| si-3 | GCAAGCAGAGCCUUAGAUCGUdTdT |
|  | ACGAUCUAAGGCUCUGCUUGCdTdT |
| RNAs for pull-down assays |  |
| NC | UUGUACUACAAAAGUACUG |
| Biotin-miR-1305 | UUUUCAACUCUAAUGGGAGAGA |
| Biotin-circCOG2 | ACAGACGAUCUAAGGCUCUGCUUGCUUCUAGUGC |
| **Abbreviations:** siRNA, small interfering RNA; qRT-PCR, quantitative real-time polymerase chain reaction; GAPDH, glyceraldehyde 3-phosphate dehydrogenase; NC, negative control. | |
