# Supplementary material for: Exosome-transmitted circCOG2 promotes colorectal cancer progression via miR-1305/TGF-β2/SMAD3 pathway
Source: Cell Death Discov. 2021 Oct 11;7:281. doi: 10.1038/s41420-021-00680-0 (PMC8505430; doi:10.1038/s41420-021-00680-0)
Supplement: Supplementary file 4 — language certificate [file 41420_2021_680_MOESM4_ESM.pdf]

MedSci

Editing By Professional Editors

CERTIFICATE OF ENGLISH EDITING

This document certifies that the manuscript entitled "Exosome-transmitted circCOG2 promotes colorectal cancer progression via miR-1305/TGF-β2/SMAD3 pathway" was proofread and edited for proper English language, grammar, punctuation, spelling, and overall style by one or more of the qualified scientific editors at MedSci, all of whom are native English speakers. Neither the research content nor the authors' intentions were altered in any way during the editing process.

Documents receiving this certification should be English-ready for publication; however, the author can accept or reject our suggestions and changes. To see the final MedSci edited version, please visit our verification page. If you have any questions or concerns about this document or certification, please contact us at editing@medsci.cn.

First Author:Lei Gao

DATE:2021-09-02

SIGNATURE:MedSci

CODE:0902-74D1-D09F-67FC-7AB2

This certificate may be verified at

https://editing.medscihealthcare.com/dist/medsci-order/#/verify
