# Supplementary material for: Exosome-transmitted circCOG2 promotes colorectal cancer progression via miR-1305/TGF-β2/SMAD3 pathway
Source: Cell Death Discov. 2021 Oct 11;7:281. doi: 10.1038/s41420-021-00680-0 (PMC8505430; doi:10.1038/s41420-021-00680-0)
Supplement: Supplementary file 5 — author contribution [file 41420_2021_680_MOESM5_ESM.pdf]

DECLARATION OF CONTRIBUTIONS TO ARTICLE

ADMC

Manuscript Number: CDDISCOVERY-21-2004-T

Journal Name: *Cell Death Discovery* (the 'Journal')

Proposed Title of the Contribution:

Exosome-transmitted circCOG2 promotes colorectal cancer progression via miR-1305/TGF-β2/SMAD3 pathway (the 'Contribution)

Author(s):

Lei Gao, Xiaolong Tang, Qingsi He, Guorui Sun, Chao Wang, Hui Qu (the 'Authors')

For all *CDDis* articles, each person named as an author in the published version must be able to show he or she has contributed substantially to the article.

Authorship credit should be based on 1) substantial contributions to conception and design, acquisition of data, or analysis and interpretation of data; 2) drafting the article or revising it critically for important intellectual content; and 3) final approval of the version to be published. Authors should meet conditions 1, 2 and 3.

Any person who cannot be shown to have made a substantial contribution to the article cannot be listed as an author in the final version. The name of any person who is deemed to have made a minor contribution can, however, appear in the Acknowledgments section of the article.

Please complete the table below to indicate the contributions of all named authors to the manuscript.

Author Full Name:	Specification of Contribution to the Manuscript:
Lei Gao	conducted the cell and animal experiments, analyzed the data and wrote the manuscript
Xiaolong Tang	conducted the cell experiments and analyzed the data
Qingsi He	collected the samples and data, and conceived and designed the experiments
Guorui Sun	collected the samples and data, and conceived and designed the experiments
Chao Wang	conducted the animal experiments
Hui Qu	conceived and designed the experiments

Please complete the table below to indicate the contributions of all named authors to the figures.

Figure 1:

Lei Gao generated the data and prepared the panel of figure 1a-g. Xiaolong Tang generated the data and prepared the panel of figure1h and 1i. Guorui Sun and Qingsi He collected the samples and collected the data. Lei Gao assembled the figure.

Figure 2:

Xiaolong Tang generated the data and prepared the panel of figure 2a-f. Lei Gao generated the data and prepared the panel of figure 2g-v. Lei Gao assembled the figure.

Figure 3:

Lei Gao generated the data, prepared the panel of figure 3a-i and assembled the figure.

Figure 4:

Xiaolong Tang generated the data and prepared the panel of figure 3a-d. Lei Gao generated the data and prepared the panel of figure 3h-j. Lei Gao assembled the figure.

Figure 5:

Lei Gao generated the data, prepared the panel of figure 5a-l and assembled the figure.

Figure 6:

Lei Gao generated the data, prepared the panel of figure 6a-n and assembled the figure.
Lei Gao and Chao Wang generated the data, prepared the panel of figure 7a-d. Lei Gao prepared the panel of figure 7e and figure S1 and assembled the figure.

Signed for and on behalf of the Author(s):	Print Name:	Date:
Hui Qu	Hui Qu	2021/8/27
